# 

**Published:** 1910-04

**Authors:** 


### CORRESPONDENCE.

### ANNUAL MEETING OF THE OREGON STATE MEDICAL ASSOCIATION.

The thirty-sixth annual meeting of the Oregon State Medical Association will be held at Portland, Wednesday, Thursday and Friday, Sept. 7, 8 and 9, 1910. These dates fall during the annual meeting of the Country Club, which celebrates with racing, exhibits and other entertainments. Visitors to the medical meeting will have an opportunity to participate in these pleasures, and will also benefit by the special railroad rates granted at that time. The Council of the Association had selected dates during the Rose Festival week in June, but before these were announced it was discovered that they would conflict with the meeting of the American Medical Association. Many of the most influential members of the State Association wish to attend the meeting of the A. M. A. and, notwithstanding that the Rose Festival would assure good attendance, it was decided the best interests of the State Association would be served by postponing the annual session until a later date. September provides a number of attractions and is sufficiently remote to warrant a second visit to the metropolis of those who attend the Rose Festival. Of these attractions, probably no other will provide better entertainment than the Country Club, and the Officers of the State Medical Association congratulate themselves on the dates selected. President Pierce intends to make the approaching meeting memorable. Negotiations are pending with noted Eastern men who are expecting to attend. No effort is being spared to secure papers of unusual merit, in which Dr. Pierce is being backed by the officers and members of the Association to the limit. If you are a member of the Oregon Association, plan to come; if you are a member of the Association of any neighboring state, come; if you are not a member of any of these, come anyway. You will be compensated for your time and trouble and you will be thrice welcome.

